# Alteration of coracoacromial ligament thickness at the acromial undersurface in patients with rotator cuff tears

**DOI:** 10.1016/j.jseint.2021.12.006

**Published:** 2022-01-27

**Authors:** Satoshi Miyake, Mikihito Tamai, Yusuke Takeuchi, Teruaki Izaki, Yasuhara Arashiro, Yozo Shibata, Terufumi Shibata, Takuaki Yamamoto

**Affiliations:** a Department of Orthopedic Surgery, Eniwa Hospital, Eniwa, Hokkaido, Japan; b Department of Orthopedic Surgery, Fukuoka University Faculty of Medicine, Fukuoka, Fukuoka, Japan; c Department of Anatomy, Fukuoka University Faculty of Medicine, Fukuoka, Fukuoka, Japan

**Keywords:** Coracoacromial ligament, Magnetic resonance imaging, Rotator cuff tear, Subacromial impingement, Acromial undersurface, Ligament thickness

## Abstract

**Background:**

Some researchers have stated that magnetic resonance imaging (MRI) is useful for assessing the coracoacromial ligament (CAL) at the acromial undersurface. However, few studies have investigated the reliability and clinical significance of MRI findings for the CAL at the acromial undersurface. The purpose of this study was to determine the association between CAL thickness at the acromial undersurface and rotator cuff tear size.

**Methods:**

The CAL thickness at the acromial undersurface was evaluated in 182 patients with rotator cuff tears (mean age: 64.9 ± 8.4 years) using a 3.0-Tesla MRI system. The association between CAL thickness at the acromial undersurface and rotator cuff tear size determined by the DeOrio and Cofield classification (partial; small: <1 cm; medium: 1-3 cm; and large or massive: >3 cm) was analyzed statistically. The intraobserver and interobserver reliabilities for MRI measurements of CAL thickness at the acromial undersurface were determined by calculation of intraclass correlation coefficients and their 95% confidence intervals.

**Results:**

The mean CAL thickness at the acromial undersurface was 2.7 ± 1.4 mm (range: 0-6.5 mm). Increasing rotator cuff tear size was significantly associated with decreasing CAL thickness at the acromial undersurface (*P* = .004). The intraobserver and interobserver intraclass correlation coefficients for CAL thickness at the acromial undersurface were almost perfect (0.98 and 0.91, respectively).

**Conclusion:**

The present study clarified that (1) MRI was a reliable tool for evaluation of CAL thickness at the acromial undersurface and (2) increasing rotator cuff tear size was significantly associated with decreasing CAL thickness at the acromial undersurface. These findings may assist toward understanding the progressive pathology in rotator cuff disease.

The coracoacromial ligament (CAL) is widely attached to the area from the anterior edge to the anterolateral site of the acromial undersurface and extends across the lateral side of the acromion.^21^ Several anatomical studies have reported variations in CAL thickness at the acromial undersurface.^10^^,^^20^ The development of imaging modalities such as ultrasound and magnetic resonance imaging (MRI) has facilitated detailed assessment of the soft tissues around joints.^2^^,^^4^, ^5^, ^6^^,^^15^^,^^31^^,^^32^^,^^34^^,^^35^^,^^39^^,^^40^ MRI is more suitable than ultrasonography for observing the soft tissues beneath bone. Researchers have suggested that MRI may be useful for CAL assessment at the acromial undersurface because the CAL is visible as a low-signal density indentation on the overlying rotator cuff on MRI images.^8^^,^^13^^,^^15^^,^^32^^,^^35^^,^^39^ Several studies involving MRI of the shoulder have shown that the CAL thickness varies at the acromial undersurface and that a thickened CAL is associated with impingement and rotator cuff tears.^8^^,^^11^^,^^13^^,^^35^^,^^36^

Progress in arthroscopic techniques has allowed detailed assessment of the acromial undersurface.^14^^,^^18^^,^^21^ Miyake et al^21^ revealed that increasing cuff tear size was significantly associated with worsening damage to the acromial undersurface, based on arthroscopic findings for the acromial undersurface in patients with rotator cuff tears. These findings were consistent with an observational study of the acromial undersurface in cadaveric shoulders conducted by Ozaki et al,^30^ who found that the severity of pathological findings at the acromial undersurface was correlated with the severity of rotator cuff tears.

The causes of rotator cuff tears have long been described as both intrinsic and extrinsic factors.^3^^,^^24^ It is true that extrinsic factors alone cannot explain the pathogenesis for the development and progression of rotator cuff tears. However, thickness alteration of the CAL at the acromial undersurface, which is representative of extrinsic factors, may be involved in the pathogenesis of rotator cuff tear progression.

The purpose of this study was to determine the association between CAL thickness at the acromial undersurface and rotator cuff tears. The hypothesis of the study was that increasing rotator cuff tear size was significantly associated with decreasing thickness of the CAL. Such information may help orthopedic surgeons to understand the pathology of subacromial impingement and to develop surgical treatment strategies for patients with subacromial impingement and rotator cuff tears.

## Methods

Radiographic data were retrospectively reviewed for 213 patients who underwent arthroscopic shoulder surgery for a rotator cuff tear at our institution between April 2016 and March 2017. Patients who had previous shoulder surgery (*n* = 1), dislocation of the shoulder (*n* = 2), calcific tendinitis (*n* = 2), frozen shoulder (*n* = 1), recurrent hemarthrosis (*n* = 1), moderate or severe osteoarthritis of the shoulder (*n* = 2), and collagen disease (*n* = 1) were excluded. Nineteen shoulders were excluded because of poor-quality radiographs. Finally, 182 shoulders in 182 patients (90 men and 92 women; mean age: 64.89 ± 8.39 years) were included in the study. The surgical procedures were performed by 3 surgeons (S.M., M.T., and Y.T.). The shoulders were divided into 4 groups as per cuff tear size based on the classification of DeOrio and Cofield^6^: partial-thickness tear (P group), 45 shoulders; small tear (<1 cm; S group), 51 shoulders; medium tear (1-3 cm; M group), 43 shoulders; and large or massive tear (>3 cm; L group), 43 shoulders, with an arthroscopic probe (in 2-mm increments). Factors potentially related to subacromial impingement syndrome (age, sex, dominant side, and acromion morphology [critical shoulder angle,^22^^,^^23^ acromial index,^26^ lateral acromial tilt,^1^ size of spurs^28^]) were statistically compared among the 4 groups.

### Measurement of CAL thickness

**Figure 1:**
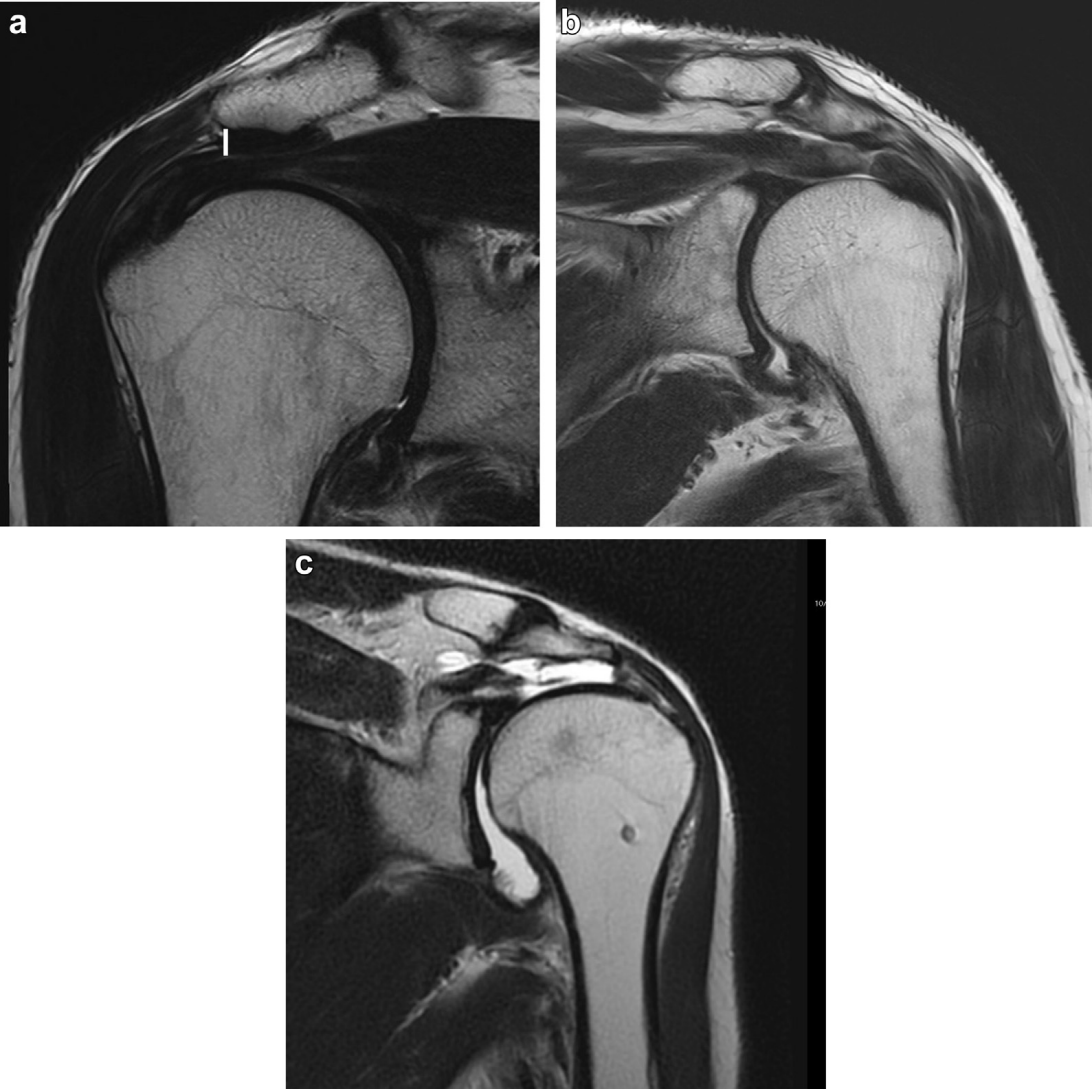
(**a**) The oblique coronal section of an MRI image showing measurement of the CAL thickness at the acromial undersurface. The CAL thickness was measured at the lateral margin of the acromion at the insertion of the CAL. A vertical line was drawn at the thickest point of the CAL insertion. (**b**) The oblique coronal section of an MRI image showing a thick subacromial CAL with a deep bursal-side partial-thickness rotator cuff tear (CAL thickness: 3.9 mm). (**c**) The oblique coronal section of an MRI image showing a thin subacromial CAL with a large rotator cuff tear and a thin subacromial CAL (CAL thickness: 0 mm). *MRI*, magnetic resonance imaging; *CAL*, coracoacromial ligament.

The MRI examinations were performed using a T2 spin echo sequence (echo time = 119; reception time = 4510) with 3-mm sections at 0.6-mm intervals, matrix size of 448×358, and field of view of 150 mm. As previously reported,^8^^,^^13^^,^^15^^,^^32^^,^^35^^,^^39^ the CAL was defined as the low-signal region on the acromial undersurface. The CAL thickness measurement was performed at the lateral margin of the acromion at the insertion of the CAL.^13^ A vertical line was drawn at the thickest point of the CAL insertion (Fig. 1).^13^

### Statistical analysis

Statistical analyses were performed using SPSS software (version 17.0; IBM, Armonk, NY, USA). First, 1-way analysis of variance, the Kruskal–Wallis test, and the chi-square test were used to assess differences in factors potentially related to subacromial impingement among the 4 groups. Second, the association between CAL thickness at the acromial undersurface and rotator cuff tear size was assessed by assigning ranks of 0-3 to a partial-thickness tear (0), small tear (1), medium tear (2), and large or massive tear (3). The Jonckheere–Terpstra trend test was used to test the association between CAL thickness at the acromial undersurface and rotator cuff tear size. *P* < .05 was considered to indicate a statistically significant difference.

To determine the intraobserver and interobserver reliabilities for subacromial CAL thickness measurements on MRI, intraclass correlation coefficients (ICCs) and their 95% confidence intervals were calculated. For intraobserver reliability, measurements of CAL thickness at the acromial undersurface were carried out twice with an interval of 1 month by 1 examiner (S.M.). For interobserver reliability, measurements of CAL thickness were carried out by 2 examiners (S.M. and Y.T.) with more than 10 years of experience. The agreement of the findings was assessed in accordance with the Landis and Koch criteria^16^ as slight (ICC: 0.00-0.20), fair (ICC: 0.21-0.40), moderate (ICC: 0.41-0.60), substantial (ICC: 0.61-0.80), and almost perfect (ICC: 0.81-1.00).

## Results

The comparisons revealed no significant differences in any of the factors potentially related to subacromial impingement among the 4 groups (Table I).

**Table I:** Relationships of patient characteristics and acromial radiographic characteristics in the rotator cuff tear groups.

	Groups				*P* value
	P	S	M	L	
Age (yr)	63.8 ± 9.1	63.4 ± 8.8	65.5 ± 8.0	67.1 ± 6.9	.13∗
Sex (male/female)	22/23	25/26	20/23	23/20	.93†
Dominant side	25	26	25	24	.92†
Critical shoulder angle (°)	34.5	34.4	34.8	35.5	.43‡
Acromion index	0.67	0.65	0.64	0.67	.06‡
Lateral acromial tilt (°)	81.4	82	81.6	79.5	.31‡
Acromial spur (mm)	1.8 ± 3.0	3.1 ± 4.6	3.4 ± 5.0	4.5 ± 5.8	.1∗

P group, a partial-thickness tear; S group, a small tear (<1 cm); M group, a medium tear (1-3 cm); L group, a large or massive tear (>3 cm).

∗ One-way analysis of variance.

† Kruskal–Wallis test.

‡ Chi-square test.

The mean CAL thickness at the acromial undersurface was 2.74 ± 1.40 mm (range: 0-6.5 mm). Regarding the sizes of the rotator cuff tears, the mean CAL thickness at the acromial undersurface was 2.89 ± 1.27 mm in group P, 3.02 ± 1.19 mm in group S, 3.13 ± 1.06 mm in group M, and 1.87 ± 1.71 mm in group L. A trend test indicated that increasing rotator cuff tear size was significantly associated with decreasing thickness of the subacromial CAL (*P* = .004).

The ICCs for the intraobserver and interobserver reliabilities for MRI measurements of CAL thickness at the acromial undersurface were almost perfect (0.98 and 0.91, respectively).

## Discussion

The present study involving MRI evaluation of shoulders in patients with rotator cuff tears revealed that (1) the mean CAL thickness at the acromial undersurface was 2.74 ± 1.40 mm and (2) increasing rotator cuff tear size was significantly associated with decreasing thickness of the CAL at the acromial undersurface (*P* = .004).

Plain X-ray and computed tomography examinations have been used to evaluate the CAL attachments at the acromial undersurface in shoulders with rotator cuff disease. These imaging modalities allow visualization of bony changes such as osteophytes,^28^ but cannot show changes in soft tissues such as ligaments. Anatomical studies that examined the CAL thickness at the acromial undersurface revealed individual variation in its thickness (range: 0-5.6 mm).^9^^,^^10^ Developments in MRI technology have made it possible to measure the CAL thickness in the shoulder of the living body.^8^^,^^13^^,^^15^^,^^32^^,^^35^^,^^39^ The present MRI observational study revealed a thickness range of 0-6.5 mm, consistent with the previous anatomical reports.^9^^,^^10^ This consistency indicates that the CAL thickness at the acromial undersurface can be adequately evaluated by MRI. However, there is still no consensus on the optimal method for MRI observation of the CAL at the acromial undersurface. To the best of our knowledge, the only study to investigate the reproducibility and accuracy of MRI measurements of the CAL at the acromial undersurface was conducted by İncesoy et al.^13^ Although there were some differences in the magnetic field strength and imaging conditions, the measurements in both studies were highly reproducible. Therefore, the CAL thickness at the acromial undersurface can be successfully measured on oblique coronal images obtained with 1.5- to 3.0-Tesla MRI systems.

The clinical significance of the CAL thickness at the acromial undersurface remains unclear, but several MRI observational studies suggested that a thickened CAL may be related to subacromial impingement and rotator cuff tears.^8^^,^^11^^,^^13^^,^^35^^,^^36^ Smith et al^35^ and Steinbach et al^36^ presented MRI images of a thickened CAL in patients with signs of impingement. Farley et al^8^ described that the prevalence of CAL thickness ≥2 mm was 38% in a supraspinatus tendon tear group, compared with 10% in an asymptomatic group. Gagey et al^11^ reported that an “aggressive” thickened CAL was found in 45% of patients with impingement, compared with 12% of patients in the control group. İncesoy et al^13^ found that the CAL at the acromial undersurface in patients with rotator cuff tears was significantly thicker than that in control patients. Taken together, these studies all suggest that the thickness of the CAL, a structure at the acromial undersurface of the shoulder, is related to rotator cuff disease.^8^^,^^11^^,^^13^^,^^35^^,^^36^

The clinical significance of the CAL thickness “alteration” at the acromial undersurface also remains unclear. The present MRI observational study is the first to show a relationship between rotator cuff tear size and CAL thickness at the acromial undersurface. This finding may reflect the process of gradual wear and tear due to repeated pathological contact between the rotator cuff and the CAL at the acromial undersurface. Routine contacts between the acromial undersurface (where the CAL is attached) and the rotator cuff were revealed in several studies.^7^^,^^12^^,^^17^^,^^19^^,^^25^^,^^29^^,^^33^^,^^41^^,^^42^ The repeated contact is considered to turn into a pathological contact termed subacromial impingement associated with shoulder motion pain in response to certain triggers. As per previous anatomical and histological studies, subacromial impingement leads to structural and histological changes (rupture) of the rotator cuff with irreversible degenerative changes, including “thickened fibrocartilaginous change of the acromial undersurface”, and finally eburnation of the acromial undersurface.^24^^,^^27^^,^^37^^,^^38^ The development of arthroscopy has allowed intraoperative assessment of changes at the acromial undersurface in patients with rotator cuff disease. Recently, Levy et al^18^ presented an arthroscopic grading system for damage to the acromial undersurface, including the CAL (Copeland–Levy classification). Miyake et al^21^ assessed the extent and degree of damage to the acromial undersurface in shoulders with rotator cuff tears, using the Copeland–Levy classification.^18^ The results showed that increasing cuff tear size was significantly associated with worsening damage to the acromial undersurface.^21^ The findings in the present MRI observational study support the previous anatomical and arthroscopic observational findings^18^^,^^21^^,^^30^ and contribute toward an understanding of the progressive pathogenesis of rotator cuff tears. However, it is not clear whether the CAL thickness “alteration” is a result of the rotator cuff tear or a cause of the rotator cuff tear. This uncertainty is similar to the case of the formation of subacromial osteophytes. To clarify this point, the CAL thickness alterations in individual patients require repeated observation by MRI for a long period.

The present findings raise questions about the predictors proposed by İncesoy et al,^13^ among which increased CAL thickness was an independent risk factor for rotator cuff tear development.^13^ These questions arise because the sizes of the rotator cuff tears included in the previous study remain unclear. The present results showed that patients with smaller rotator cuff tears had greater CAL thicknesses, but as the tear size increased, the CAL became significantly thinner. Therefore, high CAL thickness may not necessarily be a predictor of rotator cuff tear development in cases with large rotator cuff tears.

Measurement of CAL thickness at the acromial undersurface may contribute to surgical treatment strategies in patients with rotator cuff tears. Prolonged conservative therapy may be detrimental to patients with an aggressive thickened subacromial CAL with subacromial impingement signs or rotator cuff tears. In these patients, arthroscopic subacromial decompression may be considered as a treatment option. To clarify this point, prospective randomized trials focusing on CAL thickness in patients with rotator cuff tears and subacromial impingement syndrome are warranted.

The present study had several limitations. First, it was unclear whether or not the low-intensity structures at the acromial undersurface were histological ligamentous components. Second, there were no comparisons with CAL thicknesses in a healthy control group. Third, the rotator cuff tendon tear size data were not recorded accurately, although the patients were divided into 4 groups as per cuff tear size based on the classification of DeOrio and Cofield.^6^ Fourth, it was not clear whether rotator cuff tears were traumatic or nontraumatic.

## Conclusion

The present study clarified that (1) MRI was a reliable tool to evaluate CAL thickness at the acromial undersurface and (2) increasing rotator cuff tear size was significantly associated with decreasing thickness of the CAL at the acromial undersurface.

## Disclaimers

Funding: No funding was disclosed by the authors.

Conflicts of interest: The authors, their immediate families, and any research foundation with which they are affiliated have not received any financial payments or other benefits from any commercial entity related to the subject of this article.
